# Extracellular miRNA-21 as a novel biomarker in glioma: evidence from meta-analysis, clinical validation and experimental investigations

**DOI:** 10.18632/oncotarget.9188

**Published:** 2016-05-05

**Authors:** Kai Qu, Ting Lin, Qing Pang, Tian Liu, Zhixin Wang, Minghui Tai, Fandi Meng, Jingyao Zhang, Yong Wan, Ping Mao, Xiaoqun Dong, Chang Liu, Wenquan Niu, Shunbin Dong

**Affiliations:** ^1^ Department of Hepatobiliary Surgery, The First Affiliated Hospital of Xi'an Jiaotong University, Xi'an 710061, Shaanxi, China; ^2^ Department of Hematology, The Second Affiliated Hospital of Xi'an Jiaotong University, Xi'an 710004, Shaanxi, China; ^3^ Department of Hepatobiliary Surgery, The Affiliated Hospital of Qinghai University, Xining 810001, Qinghai, China; ^4^ Department of Ultrasound Diagnostics, The First Affiliated Hospital of Xi'an Jiaotong University, Xi'an 710061, Shaanxi, China; ^5^ Department of Geriatric Surgery, The First Affiliated Hospital of Xi'an Jiaotong University, Xi'an 710061, Shaanxi, China; ^6^ Department of Neurosurgery, The First Affiliated Hospital of Xi'an Jiaotong University, Xi'an 710061, Shaanxi, China; ^7^ Department of Internal Medicine, College of Medicine, The University of Oklahoma Health Sciences Center, Oklahoma City, Oklahoma 73104, USA; ^8^ State Key Laboratory of Medical Genomics, Ruijin Hospital, Shanghai Jiaotong University School of Medicine, Shanghai 200025, China

**Keywords:** extracellular miR-21, cerebrospinal fluid, glioma, diagnosis, TGF-β/Smad3 signaling

## Abstract

Evidence is accumulating highlighting the importance of extracellular miRNA as a novel biomarker for diagnosing various kinds of malignancies. MiR-21 is one of the most studied miRNAs and is over-expressed in cancer tissues. To explore the clinical implications and secretory mechanisms of extracellular miR-21, we firstly meta-analyzed the diagnostic efficiency of extracellular miR-21 in different cancer types. Eighty-one studies based on 59 articles were finally included. In our study, extracellular miR-21 was observed to exhibit an outstanding diagnostic accuracy in detecting brain cancer (area under the summary receiver operating characteristic curve or AUC = 0.94), and this accuracy was more obvious in glioma diagnosis (AUC = 0.95). Our validation study (*n* = 45) further confirmed the diagnostic and prognostic role of miR-21 in cerebrospinal fluid (CSF) for glioma. These findings inspired us to explore the biological function of miR-21. We next conducted mechanistic investigations to explain the secretory mechanisms of extracellular miR-21 in glioma. TGF-β/Smad3 signaling was identified to participate in mediating the release of miR-21 from glioma cells. Further targeting TGF-β/Smad3 signaling using galunisertib, an inhibitor of the TGF-β type I receptor kinase, can attenuate the secretion of miR-21 from glioma cells. Taken together, CSF-based miR-21 might serve as a potential biomarker for diagnosing brain cancer, especially for patients with glioma. Moreover, extracellular levels of miR-21 were affected by exogenous TGF-β activity and galunisertib treatment.

## INTRODUCTION

MicroRNAs (miRNAs) are small non-coding RNAs in 18–25 nucleotides binding to the 3′-untranslated region of massager RNA, implying a crucial important role in regulating gene expression [1, 2]. So far, there are 2588 validated human mature miRNAs according to miRBase release 21 at the website ‘http://www.mirbase.org/’. MiRNAs have been shown to play essential roles in various biological processes, and implicated as potential diagnostic and/or prognostic biomarkers in many human diseases including cancers [3]. Interestingly, a large amount of miRNA can be detected in human body fluids, including plasma, serum, urine, cerebrospinal fluid (CSF) and saliva, and they are termed as extracellular miRNAs [4]. Accumulating evidence suggests that extracellular miRNAs are exported from cells. Recently, the interest in exploring extracellular miRNAs as potential biomarkers for early cancer diagnosis and prognosis prediction is proliferating. The detection of cancer-specific extracellular miRNAs has been convincingly reported in many cancer types, including lung cancer, breast cancer, brain cancer and digestive cancers [5–7].

Recently, miR-21, as one of the most studied miRNAs, is observed to be significantly over-expressed in a broad range of cancers [8, 9] and it can serve as a potential diagnostic biomarker for cancer patients [10–14]. Besides in tissues, recent evidence indicates the presence of miR-21 in various types of extracellular fluid, such as plasma [10, 11, 15–33], serum [17, 29, 34–50], CSF [51–56], saliva [32, 57, 58], gastric juice [59], pancreatic juice [60], sputum [61], and pancreatic cyst fluid [62]. Although the diagnostic efficiency of extracellular miR-21 in cancers has been proposed by many researchers, the results are conflicting and inconclusive. To fill this gap in knowledge, we therefore designed a comprehensive meta-analysis to evaluate the diagnostic efficiency of extracellular miR-21 in various cancer types and different sample types.

In this two-phase study, we systematically investigated the expression of extracellular miR-21. In the first phase, via a comprehensive meta-analysis, we discussed the diagnostic efficiency of extracellular miR-21 in various cancer types and different sample types. Additionally, by using a validation study comprised of matched tissue and CSF samples, we evaluated the clinical significance of miR-21 as a potential diagnostic and prognostic biomarker for glioma patients. In the second phase, we examined whether cultured glioma cells can secrete miR-21 into the culture medium, aiming to establish the secretory mechanisms of extracellular miR-21 in glioma cells.

## RESULTS

### Literature search and a meta-analysis based on 81 studies

**Figure 1:**
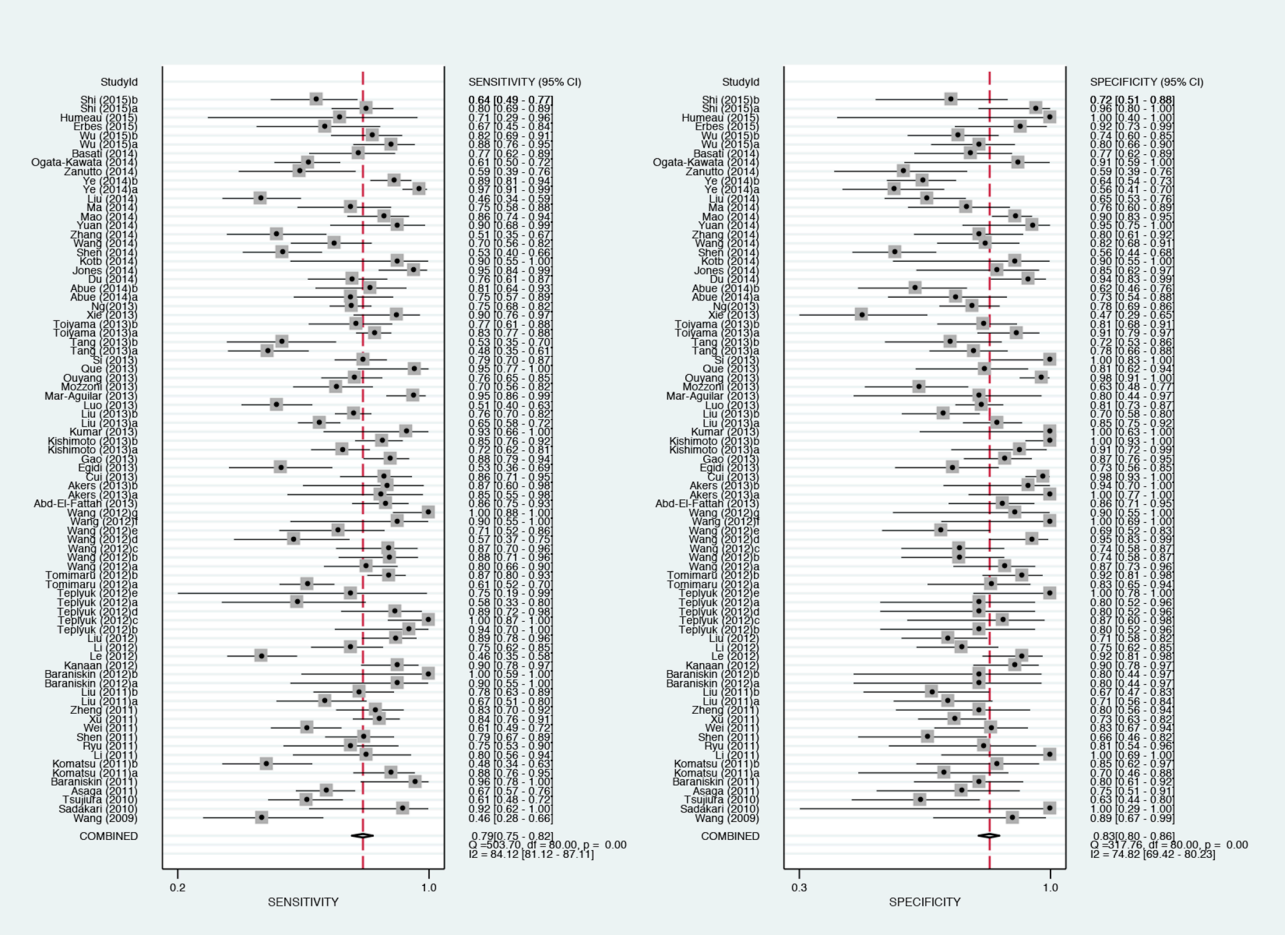
Forest plots of sensitivities and specificities for extracellular miR-21 test accuracy in cancer

In the first phase, we conducted a meta-analysis according to the guidelines set forth by the Preferred Reporting Items for Systematic Reviews and Meta-Analyses (PRISMA) statement. A flow diagram schematizing the process of article exclusion with specific reasons is presented in Figure S1. In brief, 1352 potentially relevant articles were obtained after initial search, and 1163 of them were excluded after applying further inclusion/exclusion criteria. Finally, 81 studies from 59 articles were included in this meta-analysis [10, 11, 13, 15–70]. All qualified studies recruiting 4428 corresponding cancer patients and 3066 controls were published between the year 2009 and 2015. The basic characteristics of all qualified studies are summarized in Table S1. The mostly investigated cancers included brain cancer (*n* = 14), lung cancer (*n* = 11), colorectal cancer (*n* = 11), pancreatic cancer (*n* = 9), breast cancer (*n* = 8), gastric cancer (*n* = 7), esophageal cancer (*n* = 6) and hepatocellular carcinoma (*n* = 4). Sample sources are consisted of plasma (*n* = 34), serum (*n* = 25), CSF (*n* = 12), and digestive juice (*n* = 5). Out of 81 studies, 55 were conducted in Asian populations, 20 in Caucasian populations, 2 in African populations, 1 in Caucasian & African populations and 1 in Latinos population. The meta-analysis on diagnostic accuracy of extracellular miR-21 are shown in Figure 1. After excluding outliers, overall sensitivity, specificity and area under the summary receiver operating characteristic (SROC) curve (AUC) of extracellular miR-21 for diagnosing cancers were 0.77 (0.73–0.80), 0.81 (0.79–0.84) and 0.86 (0.83–0.89) followed by their corresponding 95% confidence intervals (95%CI), respectively (Table 1).

**Table 1:** Summary estimates of diagnostic criteria and their 95% confidence intervals (95%CI) for extracellular miR-21 in cancer detection

Analysis	No. of studies	Sensitivity (95% CI)	Specitivity(95% CI)	PLR (95% CI)	NLR (95% CI)	DOR (95% CI)	AUC (95% CI)
Ethnicity							
Asian	55	0.78 (0.74–0.81)	0.83 (0.79–0.86)	4.66 (3.76–5.78)	0.26 (0.22–0.31)	18 (13–25)	0.88 (0.84–0.90)
Caucasian	20	0.80 (0.71–0.87)	0.81 (0.75–0.86)	4.29 (3.04–6.07)	0.25 (0.16–0.38)	17 (8–35)	0.87 (0.84–0.90)
Cancer sites							
Brain cancer	14	0.89 (0.80–0.94)	0.89 (0.83–0.93)	8.17 (4.95–13.47)	0.13 (0.07–0.24)	65 (25–166)	0.94 (0.92–0.96)
Breast cancer	8	0.82 (0.74–0.88)	0.86 (0.78–0.91)	5.84 (3.58–9.51)	0.21 (0.14–0.32)	27 (12–61)	0.91 (0.88–0.93)
Lung cancer	11	0.68 (0.58–0.77)	0.77 (0.69–0.84)	2.98 (2.11–4.22)	0.41 (0.30–0.56)	7 (4–13)	0.80 (0.76–0.83)
Esophageal cancer	6	0.86 (0.70–0.94)	0.65 (0.55–0.74)	2.48 (2.02–3.04)	0.22 (0.11–0.44)	11 (6–22)	0.77 (0.73–0.80)
Gastric cancer	7	0.77 (0.68–0.84)	0.85 (0.72–0.92)	5.00 (2.53–9.87)	0.27 (0.18–0.40)	19 (7–49)	0.86 (0.83–0.89)
Hepatocellular carcinoma	4	0.82 (0.70–0.90)	0.80 (0.70–0.88)	4.16 (2.63–6.59)	2.22 (0.13–0.39)	19 (8–42)	0.88 (0.85–0.90)
Pancreatic cancer	9	0.76 (0.66–0.83)	0.74 (0.67–0.80)	2.89 (2.28–3.68)	0.33 (0.23–0.47)	9 (5–14)	0.79 (0.75–0.82)
Colorectal cancer	11	0.72 (0.63–0.79)	0.83 (0.78–0.87)	4.22 (3.90–20.78)	0.34 (0.25–0.47)	12 (7–23)	0.85 (0.82–0.88)
Sample sources							
Cerebrospinal fluid	12	0.89 (0.81–0.94)	0.88 (0.82–0.92)	7.61 (5.07–11.41)	0.12 (0.07–0.22)	61 (30–126)	0.93 (0.90–0.95)
Digestive juice	5	0.80 (0.65–0.90)	0.84 (0.51–0.96)	4.97 (1.23–20.00)	0.23 (0.11–0.51)	21 (3–160)	0.88 (0.84–0.90)
Serum	25	0.79 (0.73–0.83)	0.83 (0.79–0.86)	4.55 (3.75–5.52)	0.26 (0.20–0.33)	18 (12–25)	0.88 (0.84–0.90)
Plasma	34	0.75 (0.69–0.80)	0.81 (0.76–0.85)	3.91 (2.99–5.12)	0.31 (0.24–0.39)	13 (8–20)	0.85 (0.82–0.88)
Overall	81	0.79 (0.75–0.82)	0.83 (0.80–0.86)	4.59 (3.83–5.49)	0.26 (0.22–0.30)	18 (13–24)	0.88 (0.85–0.90)
Outliers excluded	74	0.77 (0.73–0.80)	0.81 (0.79–0.84)	4.12 (3.53–4.80)	0.28 (0.24–0.33)	15 (11–19)	0.86 (0.83–0.89)

CI: confidence interval, PLR: positive likelihood ratio, NLR: negative likelihood ratio, DOR: diagnostic odds ratio, AUC: area under the curve.

Finally, Goodness of fit and bivariate normality analysis revealed that the bivariate random-effects model was robust for this meta-analysis (Figure S2A and S2B). Furthermore, we conducted an outlier detection to account for potential sources of heterogeneity. There were seven studies, 23, 35, 39, 43, 50, 57 and 71, as sources of heterogeneity for this meta-analysis. After excluding the deviated studies, there was no significant difference relative to the analysis with deviated studies (Figure S2C and S2D). We used Deek's funnel plot to evaluate publication bias of included studies. The shape of the funnel plot revealed between-study heterogeneity (*P* = 0.08, Figure S3).

### Subgroup analysis: Extracellular miR-21 as a potential biomarker in glioma

**Figure 2:**
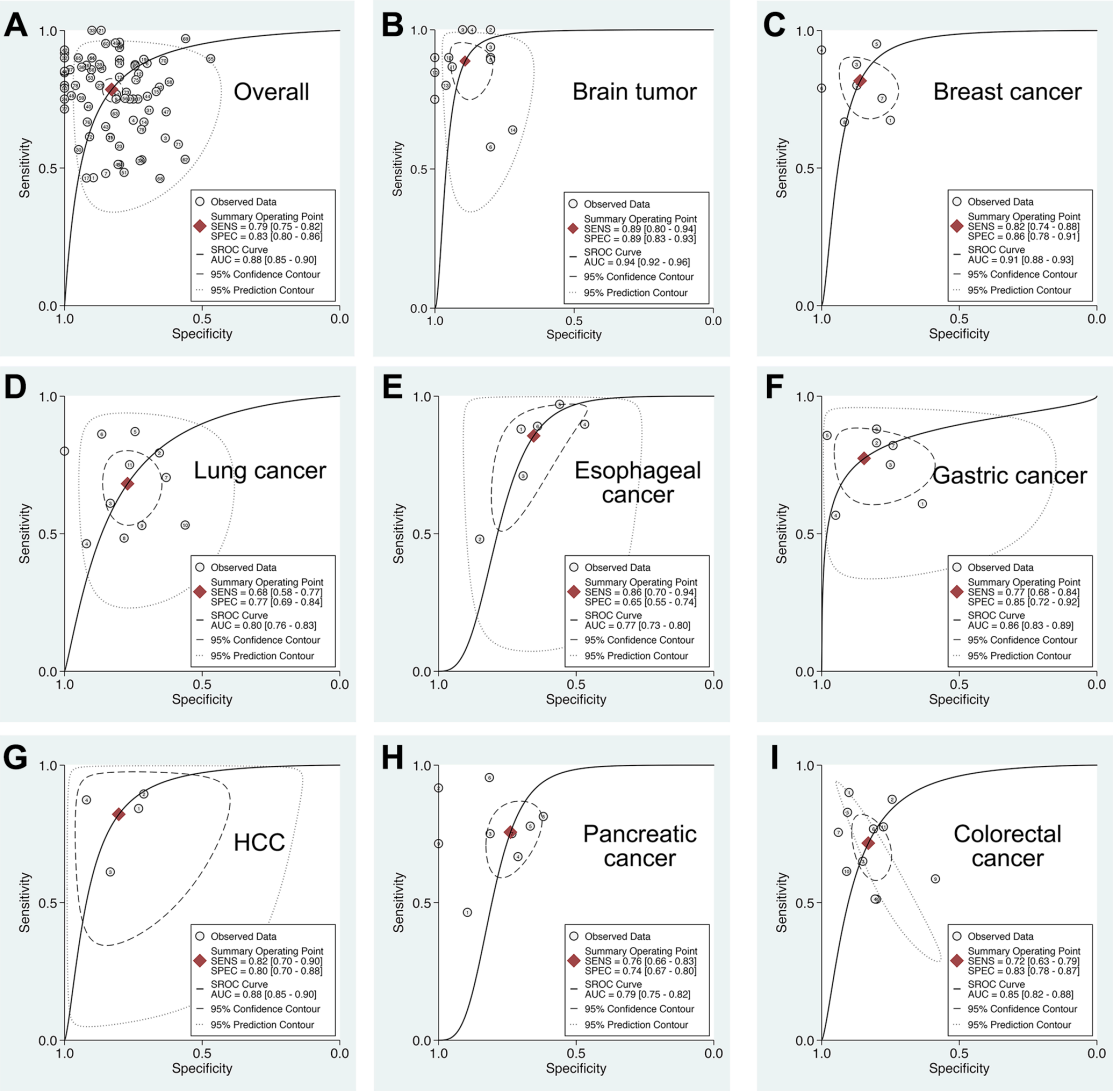
Summary ROC curve of extracellular miR-21 diagnostic values in different cancer types (**A**) Overall; (**B**) Brain tumor; (**C**) Breast cancer; (**D**) Lung cancer; (**E**) Esophageal cancer; (**F**) Gastric cancer; (**G**) Hepatocellular carcinoma; (**H**) Pancreatic cancer; (**I**) Colorectal cancer.

**Figure 3:**
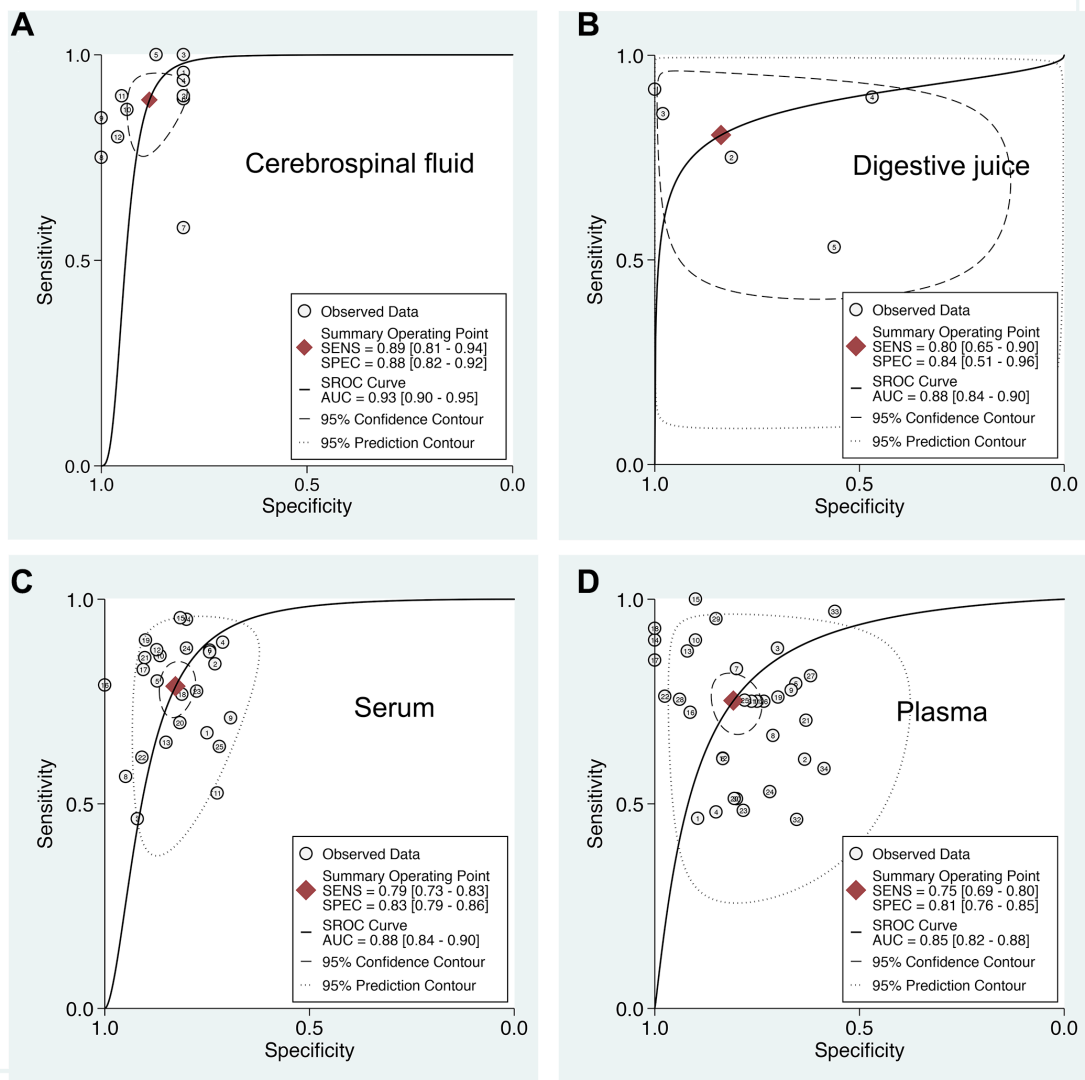
Summary ROC curve of extracellular miR-21 diagnostic values in different sample types (**A**) Cerebrospinal fluid; (**B**) Digestive juice; (**C**) Serum; (**D**) Plasma.

To account for the potential sources of between-study heterogeneity, subgroup analyses were further conducted based on ethnicity, cancer sites, and sample sources, respectively (Table 1). We found that ethnicity exerted on impact on the AUC of extracellular miR-21 (Figure S4). In contrast, the diagnostic accuracies of extracellular miR-21 varied in detecting different cancer types (Figure 2 and Table 1). Our results revealed that extracellular miR-21 had a relatively high diagnostic accuracy in detecting brain cancer, especially in detecting glioma, with a pooled AUC of 0.95 (95% CI: 0.92–0.96) (Table 2 and Figure S5). Additionally, we also found that diagnostic efficiency of extracellular miR-21for cancer differed across different sample types (Table 2 and Figure 3). Compared with other three sample types, CSF-based miR-21 detection had the highest diagnostic efficiency (sensitivity: 0.88; specificity: 0.89 and AUC = 0.94), suggesting a potential clinical role of CSF-based miR-21 in detecting patients with glioma (Figure 3). *P* values of the Deek's funnel plot for glioma and CSF subgroups were 0.41 and 0.47, respectively, indicating less likelihood of publication bias (Figure S6 and S7).

**Table 2:** Summary estimates of diagnostic criteria and their 95% confidence intervals (95%CI) for extracellular miR-21 in detection of different types of brain cancer

Variable	Subtypes of brain cancer		
	Overall	Glioma	Other brain cancers
No. of studies	14	9	5
AUC (95% CI)	0.94 (0.92–0.96)	0.95 (0.92–0.96)	0.92 (0.89–0.94)
Sensitivity (95% CI)	0.89 (0.80–0.94)	0.84 (0.73–0.91)	0.94 (0.85–0.98)
Specitivity (95% CI)	0.89 (0.83–0.93)	0.92 (0.83–0.96)	0.86 (0.75–0.92)
PLR (95% CI)	8.17 (4.95–13.47)	10.4 (4.4–24.4)	6.6 (3.7–11.8)
NLR (95% CI)	0.13 (0.07–0.24)	0.17 (0.09–0.31)	0.07 (0.03–0.19)
DOR (95% CI)	65 (25–166)	61 (16–230)	94 (27–320)

CI: confidence interval, PLR: positive likelihood ratio, NLR: negative likelihood ratio, DOR: diagnostic odds ratio, AUC: area under the curve.

### Clinical evaluation of CSF-based extracellular miR-21 level in glioma

**Figure 4:**
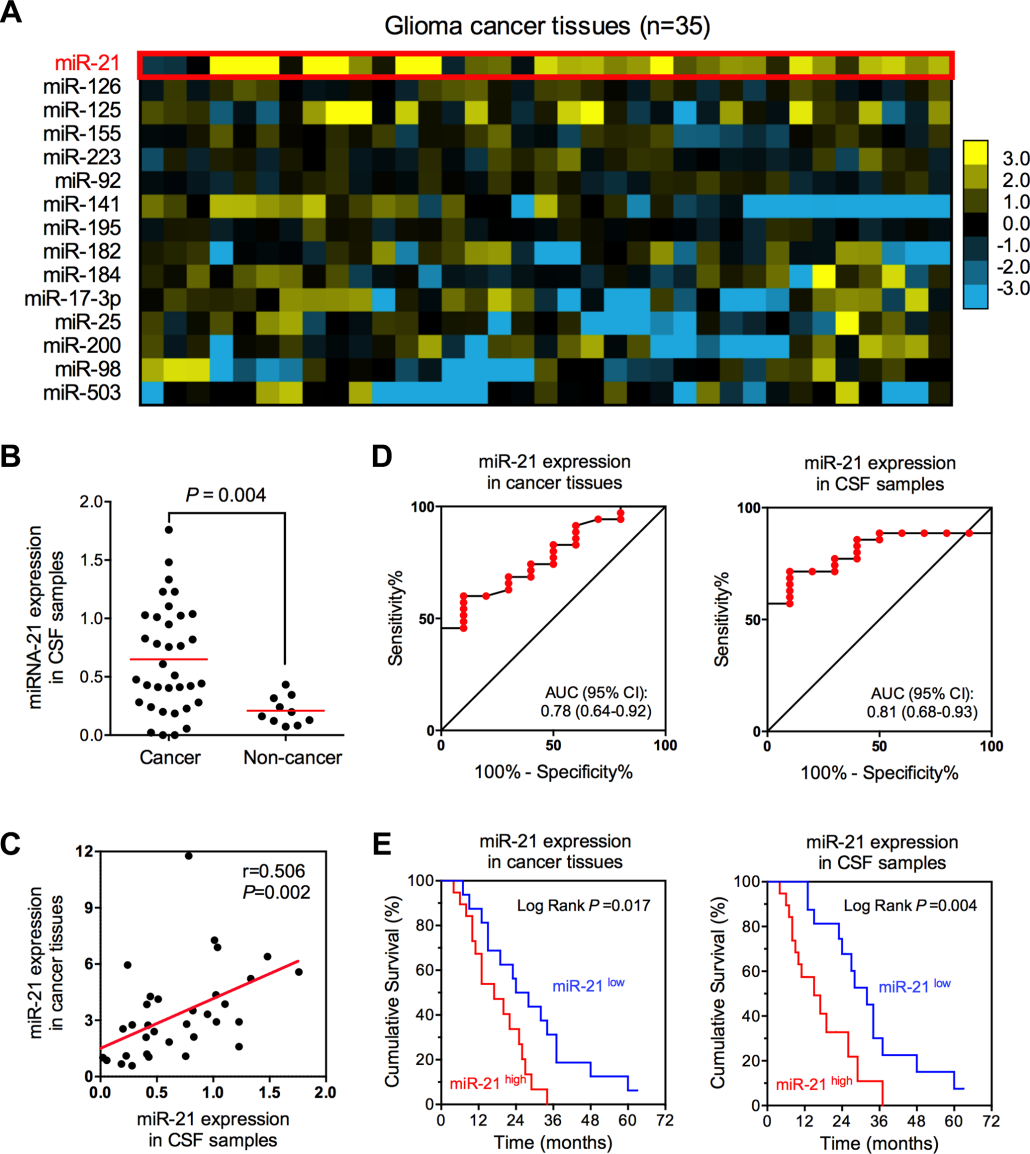
The expression of miR-21 in glioma tissue and CSF samples (**A**) Expression profile of 15 cancer-related miRNAs in glioma tissues. (**B**) CSF-based miR-21 expression in glioma patients and healthy volunteers. (**C**) Expression correlation between tissue- and CSF-based miR-21 in patients with glioma. (**D**) Diagnostic efficiencies of tissue- and CSF-basedmiR-21 in glioma. (**E**) Prognostic efficiencies of tissue- and CSF-based miR-21 in glioma.

To further evaluate the clinical potentials of miR-21 detection in glioma, we conducted a validation study by comprehensively collecting brain tissues, and paired CSF samples from 35 glioma patients and 10 non-cancer patients. The clinicopathological characteristics of 35 glioma patients are shown in Table S3. We firstly screened the expression of 15 cancer-related miRNAs in 35 glioma cancer patients (miR-125, miR-126, miR-141, miR-155, miR-17–3p, miR-182, miR-184, miR-195, miR-200, miR-21, miR-223, miR-25, miR-503, miR-92 and miR-98), which were previously reported to exist in human body fluid samples [71]. High miR-21 expression (change in expression of at least 1.5-fold when comparing the means of non-cancer tissues) was detected in 23 out of 35 (65.7%) glioma tissues (Figure 4A). Consistent with the trend in tissue samples, CSF levels of miR-21 in glioma were also significantly higher than that of non-cancer group (*P* = 0.004, Figure 4B). Moreover, we also found a strong correlation between expression levels of miR-21 in CSF samples and cancer tissues (*r* = 0.506, *P* = 0.002), indicating a close relationship between CSF and tissues expressing miR-21 (Figure 4C). Considering the high CSF-based miR-21 levels in glioma patients, we next evaluated the diagnostic accuracy of CSF-based miR-21 in glioma diagnosis. Our results showed that CSF-based miR-21 level had a high diagnostic potential in glioma diagnosis (AUC = 0.81; 95% CI: 0.68–0.93) (Figure 4D), consistent with the meta-analytical results in this study. Moreover, we found CSF-based miR-21 level also exhibited a better prognostic accuracy for glioma (Log Rank test *P* = 0.004) (Figure 4E), compared with tissue-based miR-21 level, which was previously shown to be a candidate prognostic biomarker for glioma (Figure S8, data from SurvMicro website [72]). Taken together, our data provided robust evidence for clinical implication of CSF-based miR-21 level for the diagnosis and prognosis in glioma.

### MiR-21 in extracellular environment is secreted by glioma cells

**Figure 5:**
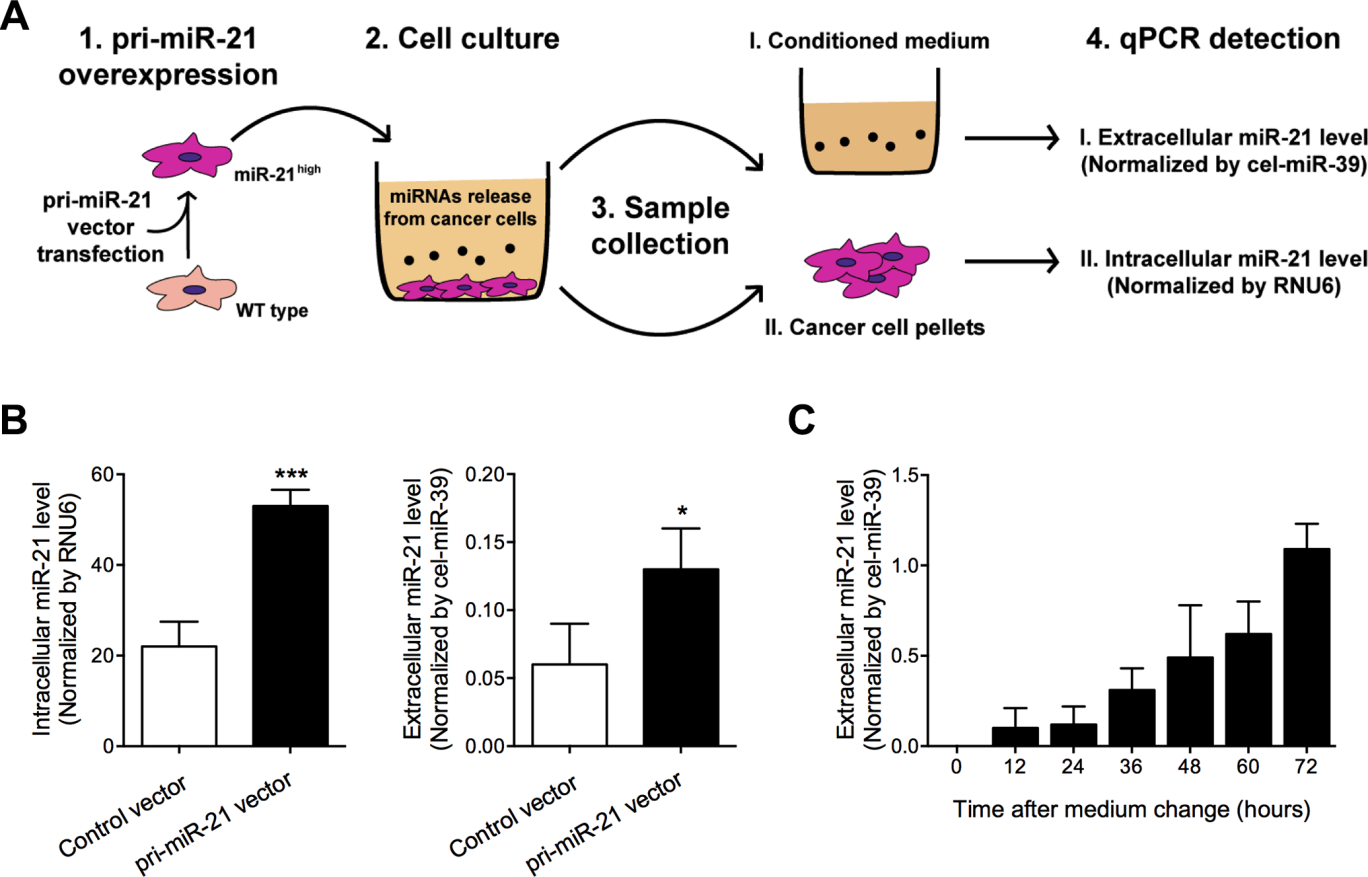
Extracellular and intracellular miR-21 levels were detected in pri-miR-21 overexpressed glioma cells after culture (**A**) Study design; (**B**) Extracellular and intracellular miR-21 levels were detected using qRT-PCR; (**C**) Extracellular miR-21 levels were detected in medium after cell culture for 0–72 hours. *P < 0.05 and ***P < 0.001.

Given the strong expression correlation between intracellular (cancer tissues) and extracellular (CSF samples) miR-21 in glioma, we speculated that extracellular miR-21 might be secreted by glioma cells which had high intracellular miR-21 expression. To test this hypothesis, we conducted a high pri-miR-21 expressing glioma cells and collected their conditioned medium for detection of extracellular miR-21 levels (Figure 5A). As shown in Figure 5B, we found that both intracellular and extracellular miR-21 were increased in pri-miR-21 vector transfected glioma cells. Moreover, we also found that the extracellular miR-21 levels in culture medium followed a time-dependent manner (Figure 5C), further suggesting the secretory process of miR-21 in glioma cells.

### TGF-β signaling induced miR-21 expression in glioma cells

**Figure 6:**
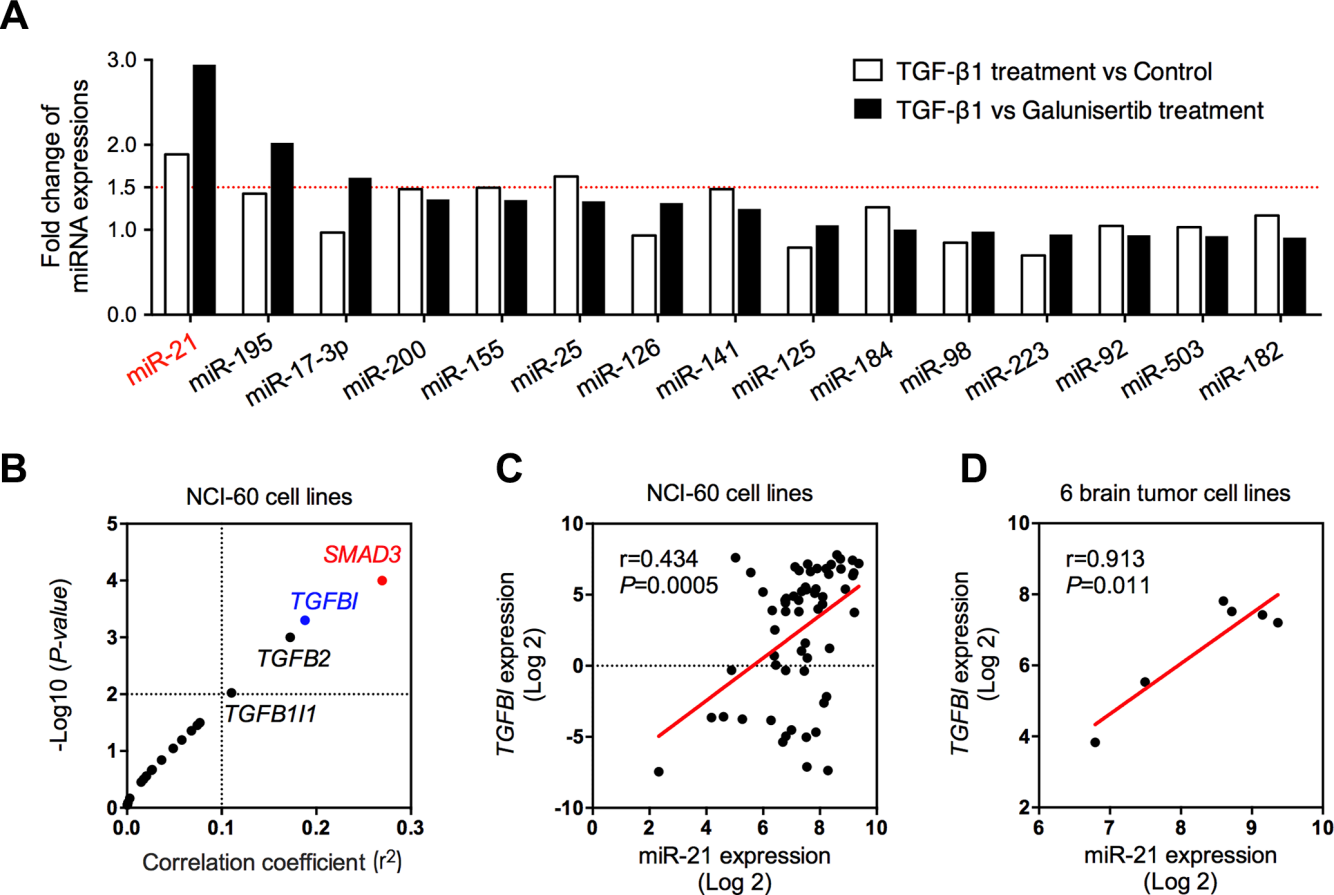
Association between miR-21 and TGF-β signaling pathway (**A**) The expression changes of 15 cancer-related miRNAs after TGF-β1 treatment, comparing with control or TβR inhibitor (galunisertib) treatment group; (**B**) Expression correlation between miR-21 and genes in TGF-β signaling pathway in 60 cancer cell lines; (**C**) Expression correlation between miR-21 and *TGFBI* in 60 cancer cell lines; (**D**) Expression correlation between miR-21 and *TGFBI* in 6 brain tumor cell lines.

Previous studies suggested that TGF-β pathway involved in intracellular regulation of miR-21 [73, 74]. However, the corresponding molecular mechanisms remains unclear. In this study, we found that addition of extracellular TGF-β1, the most powerful activator of TGF-β signaling, can significantly induce intracellular miR-21 expression (Figure 6A). Additionally, the fold change of intracellular miR-21 was shown to be much higher (approximately three-fold increase) in U251 cells receiving TGF-β1, when compared with those cells under galunisertib treatment (a selective inhibitor of TGF-β signaling [75]), suggesting an association between intracellular miR-21 and TGF-β signaling (Figure 6A).

To further explore the factors mediating TGF-β-miR-21 pathway, we evaluated the expression correlation between 19 genes in TGF-β signaling pathway and miR-21 expression using NCI-60 expression profiling data (GSE5846 and GSE26375). TGFBI, a secreted protein induced by TGF-β, has been considered to be tightly associated with extracellular levels of TGF-β [76]. We therefore selected *TGFBI* as a mediator to see the relationship between extracellular TGF-β levels and intracellular miR-21 expression. As demonstrated in Figure 6B, *TGFBI* was one of the four top genes in close correlation with miR-29b expression. The expression association between *TGFBI* and miR-21 was shown to be significant with a *P* value of lower than 0.05 either in the NCI-60 cells (Figure 6C) or in 6 brain cancer cell lines (including SF-268, SF-295, SF-539, SNB-19, SNB-75 and U251 cell lines, Figure 6D). Above data once again confirmed the inductive activity of TGF-β in intracellular miR-21 expression.

### Secretion of miR-21 in glioma cells depends on Smad3 activity

**Figure 7:**
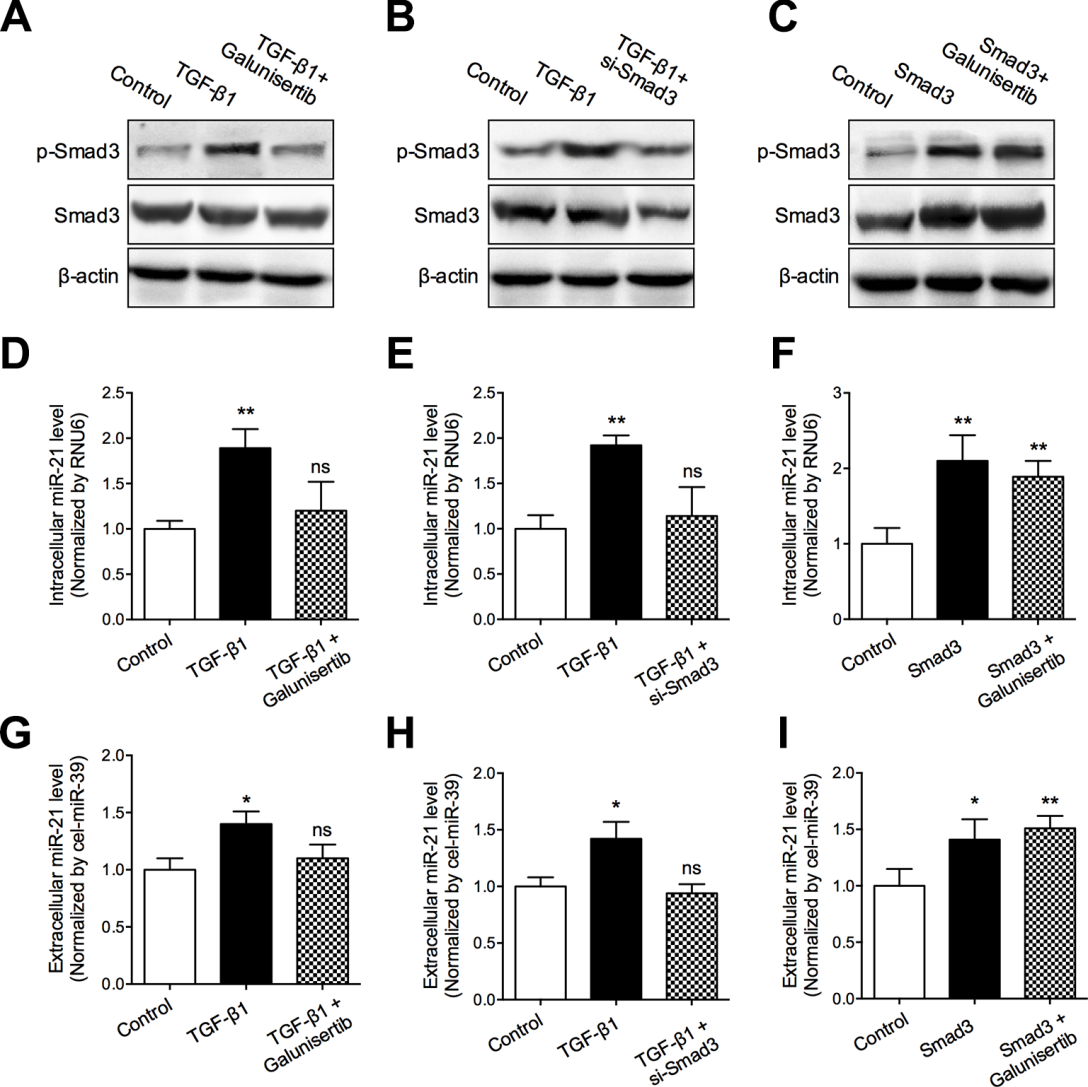
TGF-β/Smad3 signaling pathway mediated miR-21 secretion (**A**–**C**) Smad3 phosphorylation status in the corresponding subgroups were detected using Western blotting assay; (**D**–**F**) Intracellular miR-21 levels in the corresponding subgroups were detected using qRT-PCR assay; (**G**–**I**) Extracellular miR-21 level in the corresponding subgroups were detected using qRT-PCR assay. *P < 0.05 and **P < 0.01.

As demonstrated in Figure 6B, we found that *SMAD3* was significantly associated with intracellular miR-21 expression. Previous studies suggested that Smad3 was identified as key mediator for transducing TGF-β signals from the cell membrane to the nucleus, and was highly expressed in the brain. Therefore, to Figure out whether activated Smad3 was involved in production and secretion of miR-21, we detected the intracellular and extracellular levels of miR-21 in different activation status of Smad3. Our data showed that TGF-β1 induced phosphorylation of Smad3 and increased intracellular and extracellular levels of miR-21. In contrast, galunisertib significantly inhibited the phosphorylation of Smad3 induced by TGF-β1 and depressed miR-21 levels (Figure 7A, 7D and 7G). When blocking Smad3 activity using small interference RNA (siRNA) of Smad3, the intracellular and extracellular levels of miR-21 were significantly decreased, compared with TGF-β1 treated cells (Figure 7B, 7E and 7H). Furthermore, we also investigated the effect of Smad3 over-expression on miR-21 levels. We found that transfection of Smad3 expressing vector increased the intracellular and extracellular levels of miR-21, which was not inhibited by galunisertib treatment (Figure 7C, 7F and 7I). Above results suggested that Smad3 activity was essential for production and release of miR-21 induced by TGF-β1 in glioma cells.

## DISCUSSION

In this two-phase study, we systematically investigated the expression of extracellular miR-21. In the first phase, by conducting a comprehensive meta-analysis, we discussed the diagnostic efficiency of extracellular miR-21 in various cancer types and different sample types. Additionally, by using a validation study comprised of matched tissues and CSF samples, we evaluated the clinical significance of miR-21 as a potential diagnostic and prognostic biomarker for glioma patients. In the second phase, we demonstrated that cultured glioma cells secreted miR-21 into extracellular medium, and TGF-β/Smad3 signaling pathway was involved in the secretory regulation of miR-21 in glioma cells.

Recent studies in growing numbers have shown that extracellular miRNAs might be a new potential biomarker for early diagnose of cancers [5, 6]. MiR-21, one of the most studied miRNAs, has been widely reported for its over-expression in various cancer types, and has also been found in extracellular environment [10, 12–14, 77]. In the present study, we performed a meta-analysis of 7494 participants derived from 81 published studies worldwide. Compared with other published meta-analyses involving diagnostic accuracy of extracellular miR-21 [78–82], our analysis included totally 81 studies that covered multiple cancer types and sample types, and therefore provided more comprehensive and innovative results. To the best of our knowledge, this is the largest meta-analysis exploring evaluate the diagnostic efficiency of extracellular miR-21 in cancer diagnosis. Our meta-analysis demonstrated that the pooled sensitivity, specificity and AUC of extracellular miR-21 in cancer diagnosis was 0.79, 0.83 and 0.88, respectively, indicating a high accuracy in diagnosing cancers. Interestingly, we further found that miR-21 showed an outstanding efficiency in diagnosis of brain cancer, with a sensitivity of 0.89, specificity of 0.89, and AUC of 0.94. In glioma, the pooled AUC of extracellular miR-21 was increased to 0.95. In addition, we found that sample sources also exerted an impact on the diagnostic accuracy of extracellular miRNA-21. Our results showed that CSF-based miR-21 had a much higher efficiency than miR-21 derived from other sample types, with sensitivity of 0.88, specificity of 0.89, and AUC of 0.94. Malignant glioma has a high mortality with no curative therapies available. Thus, it is urgent to find novel biomarkers for the early diagnosis of glioma. In contrast to blood samples, the miRNA profiles of CSF are mainly affected by CNS diseases and are less affected by blood miRNA concentrations due to blood-brain barrier. Considering above clear strengths, CSF sample is more predictable for real-time monitoring cancer burden and therapeutic response in patients with glioma. Therefore, to evaluate the diagnostic accuracy of CSF-based miR-21 in glioma, we further conducted a validation cohort recruiting 35 glioma patients and 10 non-cancer patients. Our validation results confirmed the findings that CSF-based miR-21 had a high diagnostic accuracy in glioma diagnosis, with an AUC of 0.81 [52]. Furthermore, in our validation cohort, we also found that CSF-based miR-21 exhibited a higher accuracy than tissue-based miR-21 for prognostic prediction of glioma, consistent with a previous report [56]. Taken together, our meta-analysis and clinical validation strongly suggested the potential application of CSF-based miR-21 in glioma, and large-scale multicenter studies are warranted to confirm or refuse our findings.

Despite the strong association between CSF-based miR-21 and glioma, it has not been investigated whether or not the miR-21 existed in CSF was secreted from glioma cells. Previous studies have clearly demonstrated high intercellular miR-21 expression in glioma cells *in vitro* and *in vivo* [83–87]. Recently, Baraniskin A et al. firstly observed an increased miR-21 level in CSF samples from patients with glioma [52]. Shi et al. further identified that miR-21 existed in CSF derived exosomes, suggesting that CSF-based miR-21 might be secreted by glioma cells. In this study, by conducting a high miR-21 expression glioma cell, we investigated the secretion of miR-21 in glioma cells. Our data showed that the relative extracellular miR-21 level elevated in a time-dependent manner, which provided evidence for the secretory process of miR-21 from glioma cells into extracellular environment.

**Figure 8:**
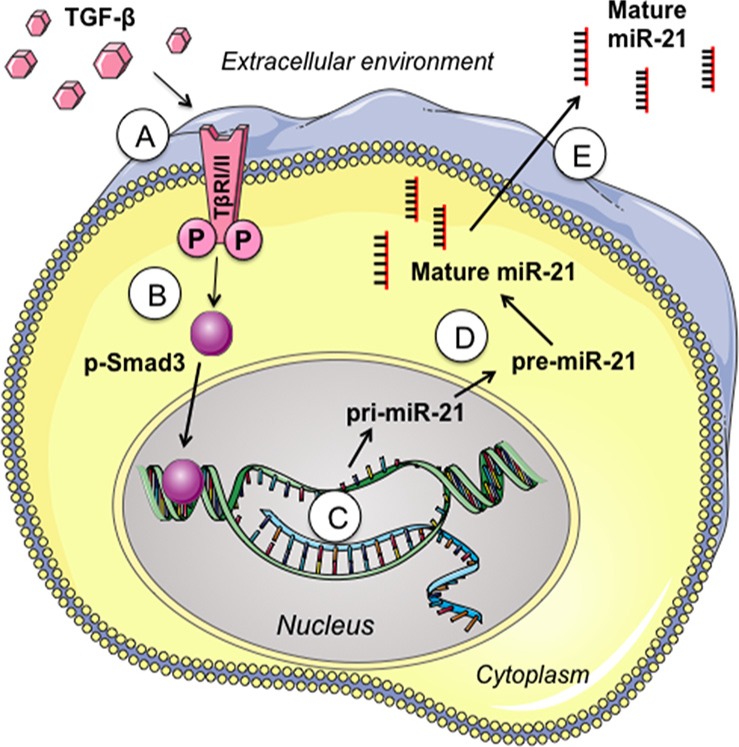
Overview of TGF-β/Smad3 signaling pathways in regulating miR-21 secretion (**A**) Exogenous TGF-β binds to TGF-β receptors (TβRI/II); (**B**) Activated TGF-β receptors phosphorylate and thus activate the intracellular signaling mediator Smad3; (**C** and **D**) Phosphorylated Smad3 promotes the processing and maturation of pri-miR-21; (**E**) Mature miR-21 is secreted into extracellular environment.

TGF-β signaling pathway has gained a special interest in recent years because of its essential roles in regulating cell growth, proliferation, differentiation, and apoptosis. Previous studies have suggested an association between TGF-β signaling pathway and miR-21 [73]. Recently, it is reported that TGF-β1 is capable of inducing numerous miRNAs, including miR-21, in renal fibrosis and lung cancer [88, 89]. Wang et al. [90] also observed the expression correlation between TGF-β and intracellular miR-21 in ursolic acid-treated U251 cells. However, it was still uncertain whether or not TGF-β signaling pathway was involved in miR-21 secretion in glioma. In this study, we clearly demonstrated that both intracellular and extracellular expressions of miR-21 were induced by TGF-β. Meanwhile, inhibition of TGF-β signaling pathway using specific TGF-β receptor inhibitor can significantly suppress both intracellular and extracellular miR-21 expression. To explore the detailed secretory mechanisms of miR-21 in glioma, we further screened the expression correlation between miR-21 and 19 members of TGF-β signaling pathway. Our data revealed that Smad3, one of the most important mediators of TGF-β signaling pathway, was strongly associated with miR-21 expression. Furthermore, we also found that over-expressed Smad3 induced secretion of miR-21 from glioma cells into extracellular environment. Meanwhile, inhibition of Smad3 attenuated the TGF-β induced miR-21 secretion. Above findings provided evidence for the essential roles of TGF-β/Smad3 signaling pathway in mediating miR-21 secretion in glioma (Figure 8).

Despite the clear strengths including a meta-analysis based on a large number of studies and independent validation using clinical and experimental data, several possible limitations in our study should be noted. First, we only conducted a meta-analysis to investigate extracellular miR-21 in glioma diagnosis, but did not meta-analyze its prognostic potentials in survivorship of glioma patients, mainly due to the lack of literature on this topic. Second, because of the small sample size recruited in our study, tissue and CSF-based miR-21 expression was compared between glioma patients and healthy volunteers, and we failed to take different tumor origin, tumor stages and histological classification into consideration. Third, in the experimental validation, we only provided evidence that TGF-β/Smad3 signaling pathway is essential in regulating miR-21 secretion, but the detailed mechanisms involving TGF-β/Smad3 mediated miR-21 production and secretion is still unknown and warrants further investigation.

In conclusion, by conducting meta-analysis, clinical and experimental investigations, we demonstrated that extracellular miR-21 level, especially CSF-based miR-21 level, could serve as a potential biomarker for glioma patients. Our results further suggest that CSF-based miR-21 might be secreted by glioma cells and TGF-β/Smad3 signaling pathway was responsible for regulating miR-21 secretion.

## MATERIALS AND METHODS

### Search strategy

A literature search for studies that explored the diagnostic value of circulating miR-21 for cancers was conducted among several computerized databases, including PubMed, Embase, Google Scholar, Cochrane Library, Scopus, Chinese Biomedical Literature Database (CBM) and Chinese National Knowledge Infrastructure (CNKI). The search terms we used were as follows: [“microRNA-21” or “miRNA-21” or “miR-21”] AND [“cancer” or “carcinoma” or “neoplasms” or “tumor”] AND [“sensitivity” or “specificity” or “ROC curve” or “diagnosis”]. Additionally, citations in retrieved articles as well as systemic reviews or meta-analysis on the same topic were also searched where relevant.

### Inclusion and exclusion criteria

Three investigators (Kai Qu, Tian Liu and Wenquan Niu) independently scanned the titles and abstracts of all retrieved articles to evaluate their eligibility. The articles fulfilling the following criteria were included for further analyses: (1) investigation of the diagnostic potential of circulating (blood, serum, plasma, digestive juice and CSF) miR-21 for human cancers; (2) the diagnosis of cancer patients was confirmed by pathological detection; (3) studies provided sufficient data, including case and control number, sensitivity and specificity. The exclusion criteria were as follows: (1) studies were obviously unrelated to diagnostic value of circulating miR-21; (2) studies were in forms of letters, editorials, case reports, meta-analyses or reviews.

### Data extraction and meta-analysis

Data from all eligible studies were extracted as followings: basic characteristics of articles (including author name, publication year, country of the study, ethnicity, number of cases and controls, cancer and sample types), and diagnostic results [including sensitivity, specificity, AUC, true positive (TP), true negative (TN), false positive (FP), and false negative (FN)], respectively. All the meta-analysis were carried out using above data by the STATA12.0 software. To evaluate diagnostic effects, bivariate meta-analysis models were employed to calculate the pooled sensitivity, specificity, PLR, NLR, DOR and AUC. The summary receiver operator characteristic (ROC) curve was also conducted by pooling the sensitivity and specificity of each study. We performed Deek's funnel plot to explore whether there was publication bias in our included studies; the *P* value was set at 0.10, which meant if *P* < 0.10, publication bias was significant.

### Patients and sample collection

Overall, 35 patients pathologically diagnosed with glioma were included in the study. All included individuals were Chinese Han People and were recruited from the First Affiliated Hospital of Xi'an Jiaotong University. All tumor specimens obtained intra-operatively were collected, and were immediately fresh-frozen on dry ice and stored at −80°C until further detection. At least 2 mL of CSF sample was collected from each patient. All CSF samples were immediately cleared of cells and debris after collection by brief centrifugation and then stored at −80°C. The clinical-pathological characteristics of all participants recruited in the study are summarized in Table S2. This study has been approved by the Institutional Review Board for Human Research of the First Affiliated Hospital of Xi'an Jiaotong University.

### Cell culture and reagents

Human glioma cell line U251 was obtained from Shanghai Institute of Biochemistry and Cell Biology, Chinese Academy of Sciences (Shanghai, China). U251 cells (5.0 × 10^4^ cells/mL) were cultured in DMEM medium supplemented with 10% fetal calf serum (FCS) and penicillin/streptomicyn in a humidified atmosphere of 5% CO_2_/95% air at 37°C. Cell pellets and conditioned medium were harvested every 12 h during cell culture and stored at −80°C for further detection.

TGF-β1 was purchased from R&D Systems (Minneapolis, MN) and was dissolved at working concentration of 5.0 ng/ml. Galunisertib (TGFβ receptor I kinase inhibitor, LY2157299) was purchased from Selleck Chemicals (Houston, TX, USA) and was dissolved in PBS to make a 50 mmol/L stock solution. All above reagents were stored at −20°C.

### Generation of miR-21 stably overexpressing cells

The expression plasmid for miRNA-21 (pCMV -miR-21) and the corresponding empty vector were purchased from OriGene (Rockville, MD). U251 cells were transfected with pCMV-miR-21 vector or empty vector in 24-well dishes using Lipofectamine reagent (Invitrogen, Carlsbad, CA) according to the manufacturer's protocol. After 24 h transfection, the cells were replaced in a 10-cm dish followed by a 14-day selection using G418 (1 mg/ml). Surviving colonies were picked up from each transfectant and were then cultured for another 2 weeks in the presence of 0.3 mg/ml G418. The cells expressing the largest amount of pCMV-miR-21 were used as miR-21 stably expressing cells.

### Smad3 siRNA and expression vector transfection

The siRNA sequences corresponding to the cDNA sequences of Smad3 (NM_005902) was as following: Smad3 siRNA, 5′-TCCGCATGAGCTTCGTCAA-3′; scramble negative control (SNC) siRNA, 5′-TTCTCCGA ACGTGTCACGTTT-3′. Smad3 expression vector (pMD-SMAD3) was purchased from Sino Biological Inc (Beijing, China). Equal amounts of an empty vector pcDNA3.1 serve as control. Transfection of siRNA (100 nM) or plasmid (4 μg) was carried out using a Lipofectamine 2000 reagent (Invitrogen, Carlsbad, CA) according to the procedure recommended by the manufacturer.

### Total RNA extraction and quantitative real-time PCR

Total RNA derived from tumor tissues and cultured cells was extracted using Trizol Reagent (Invitrogen, Carlsbad, CA, USA), and total RNA derived from CSF and medium were isolated using Trizol LS Reagent (Invitrogen, Carlsbad, CA, USA) according to the manufacturer's protocol. As previously reported [91, 92], miRNA expression levels were determined by quantitative real time-PCR applying the SYBR^®^ PrimeScriptTM miRNA RT-PCR Kit and SYBR^®^ Premix Ex TaqTM (TaKaRa Biotechnology, Dalian, China). The miRNA expression was assayed in triplicate and normalized to corresponding housekeeping miRNAs, RNU6 (for intracellular level of miRNAs) and cel-miR-39 (for extracellular level of miRNAs). The relative miRNA levels were calculated using the Comparative-Ct method (Ct method). All primers were synthesized by TaKaRa Biotechnology.

### Western blotting

Cultured cells were lysed in RIPA buffer (Beyotime Inc., NanTong, China). Protein concentration was identified using the Bradford reagent (Beyotime Inc.). We then performed electrophoresis of protein extracts and subsequent blotting as previously described [91, 92]. In brief, Equivalent amounts of protein (30 μg) were separated by SDS-PAGE gels and transferred to polyvinylidene difluoride (PVDF) membranes (Bio-Rad, Hercules, CA, USA). The membranes were then immunoblotted with the appropriate primary antibodies against Smad3 or p-Smad3 (Santa Cruz Biotechnology, Santa Cruz, CA), at 4°C for overnight, and subsequently were incubated with HRP conjugated anti-mouse or anti-rabbit secondary antibodies at room temperature. Signals were detected using an enhanced chemiluminescence (ECL) system (Beyotime, China) on Kodak X-ray film. Equal protein loading was assessed by the expression of β-actin. The protein bands were quantified using the BioRad Quantity One software package.

### Statistical analysis

All experiments in this study were repeated three times. The data were analyzed by SPSS 11.0 software (SPSS Inc, Chicago, IL, USA) and expressed as mean ± standard error of measurement (SEM). Analysis of continuous variables were performed using a Student's *t*-test and *P* < 0.05 was considered statistically significant.

## SUPPLEMENTARY FIGURES AND TABLES




