# Supplementary material for: Extracellular miRNA-21 as a novel biomarker in glioma: evidence from meta-analysis, clinical validation and experimental investigations
Source: Oncotarget. 2016 May 5;7(23):33994–4010. doi: 10.18632/oncotarget.9188 (PMC5085133; doi:10.18632/oncotarget.9188)
Supplement: Supplementary file 1 [file oncotarget-07-33994-s001.pdf]

Extracellular miRNA-21 as a novel biomarker in glioma: evidence from meta-analysis, clinical validation and experimental investigations

Supplementary Materials

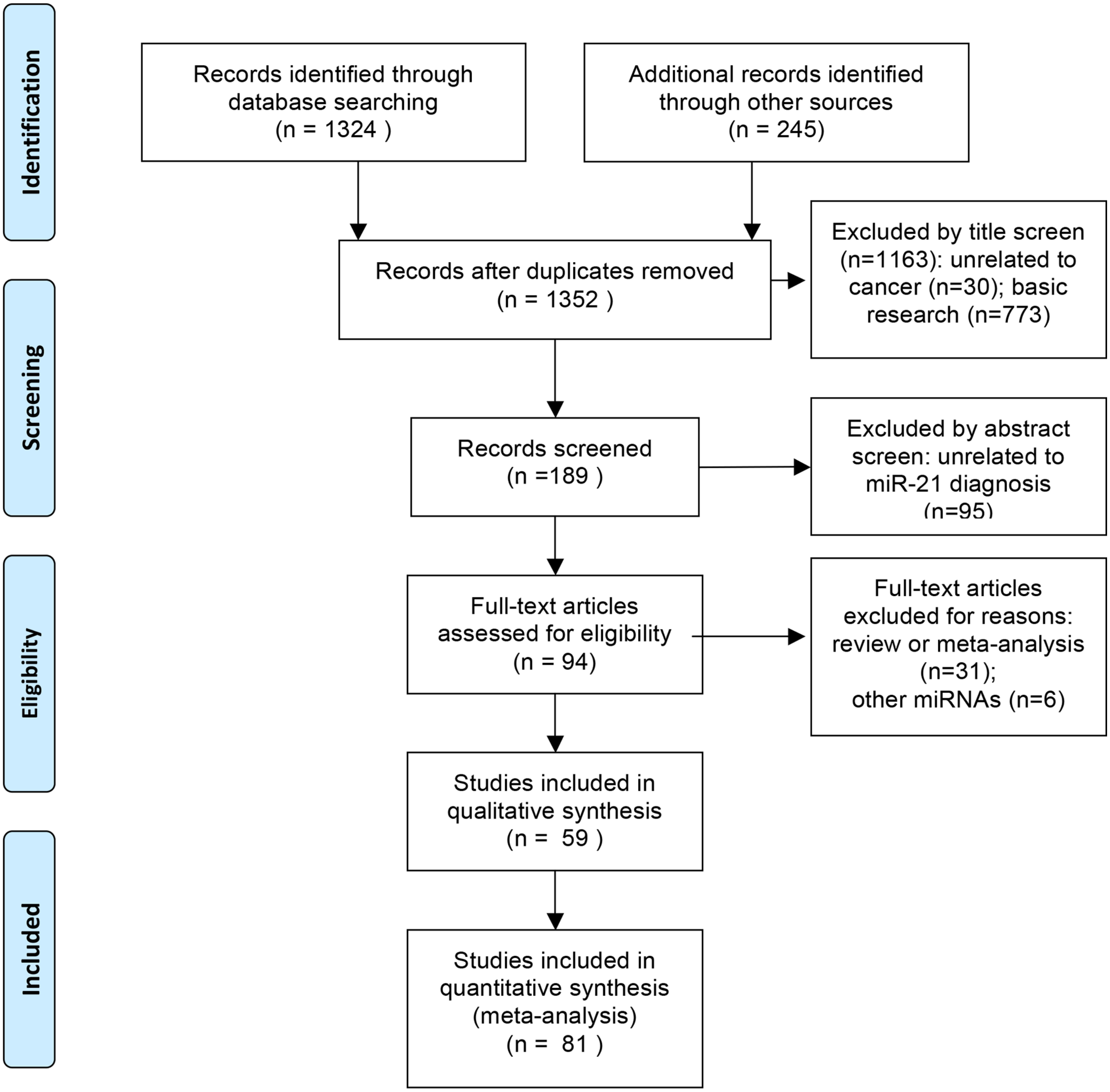

Supplementary Figure S1: Flow diagram of the selected eligible studies based on the inclusion and exclusion criteria.

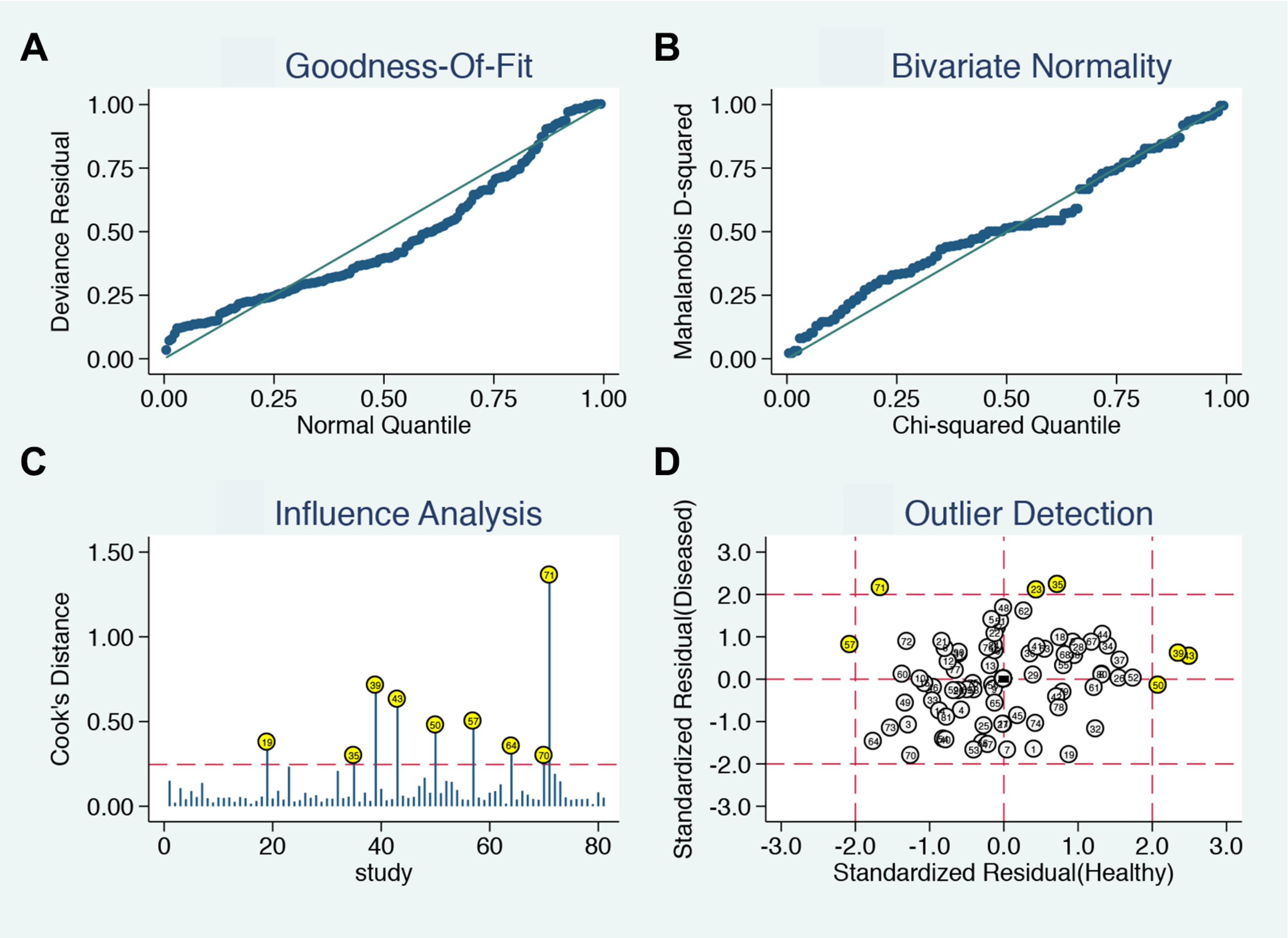

Supplementary Figure S2: Sensitivity analysis (A) goodness-of-fit; (B) bivariate normality; (C) influence analysis; (D) outlier detection.

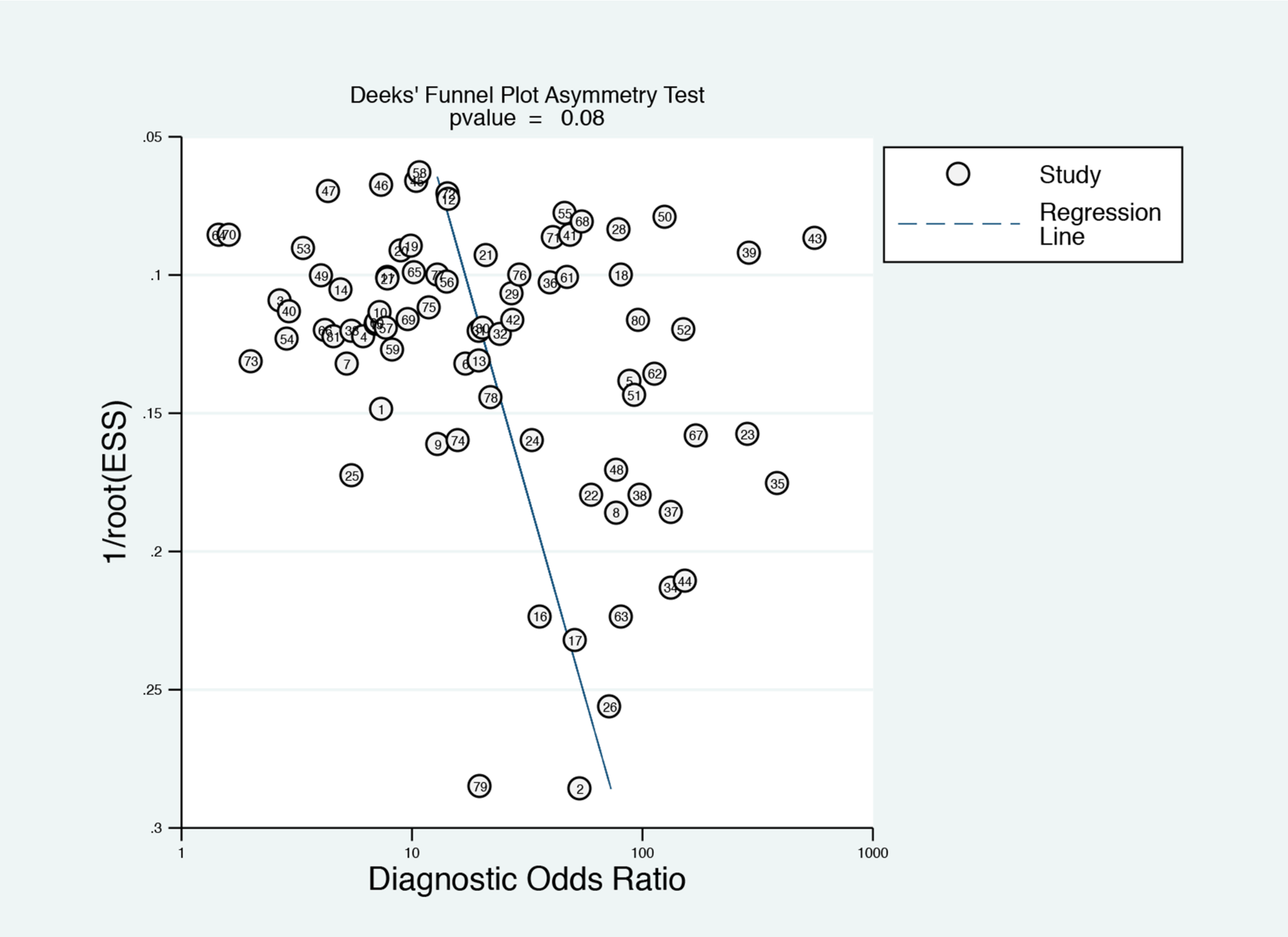

Supplementary Figure S3: Funnel graph for the assessment of potential publication bias for overall studies.

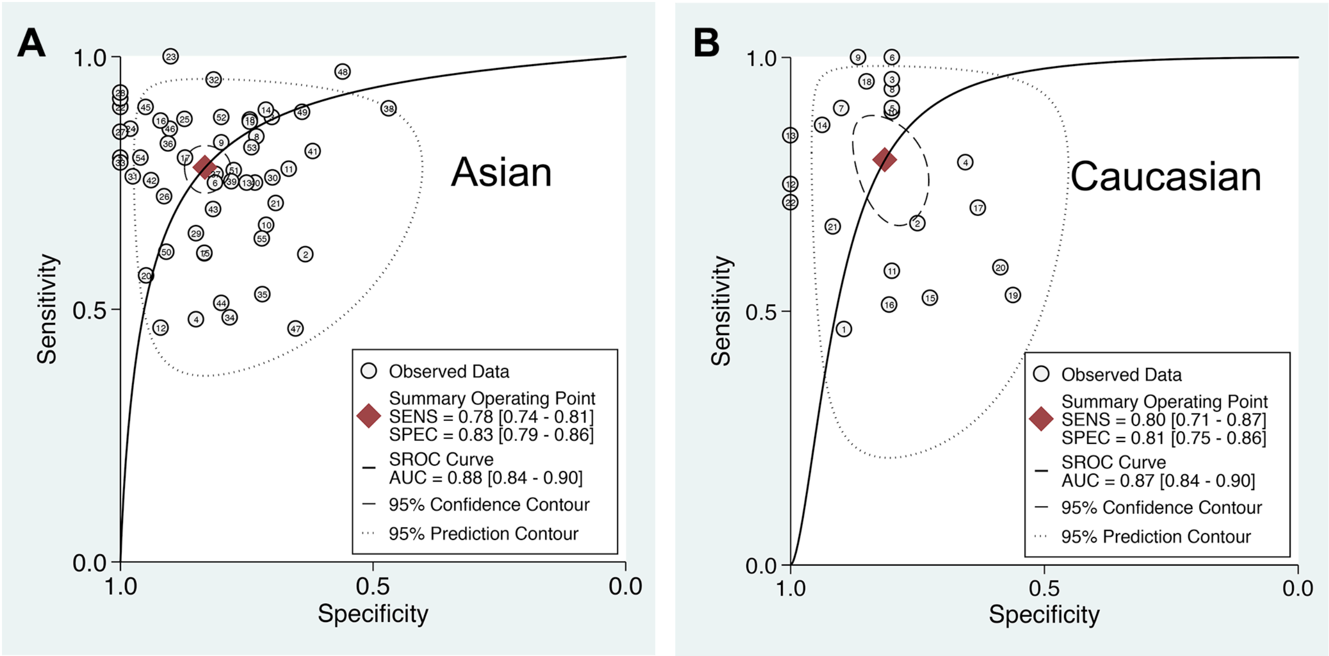

Supplementary Figure S4: Summary ROC curve of extracellular miR-21 diagnostic values in different ethnic population. (**A**) Asian; (**B**) Caucasian.

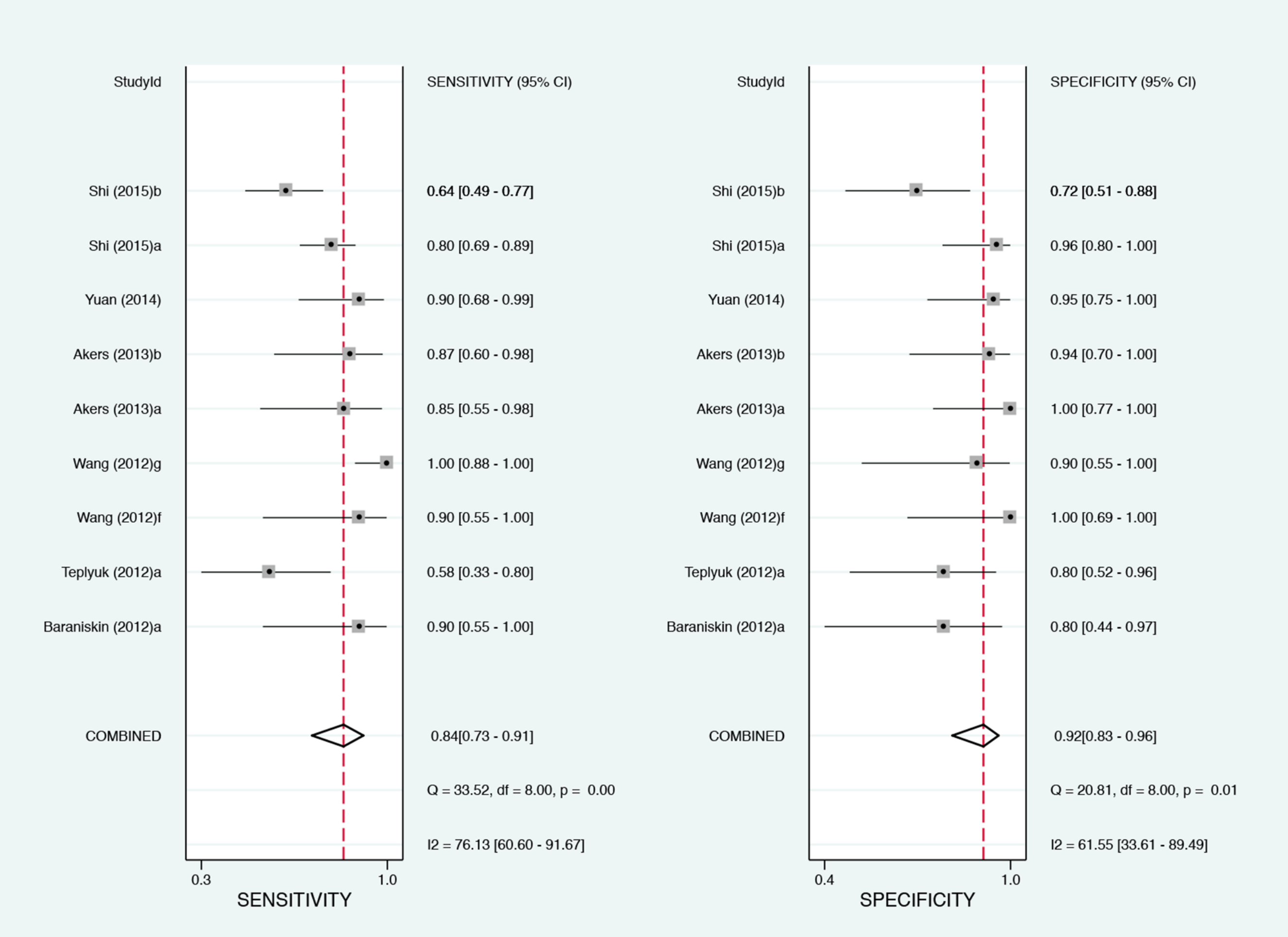

Supplementary Figure S5: Forest plots of sensitivities and specificities for CSF-based miR-21 test accuracy in glioma.

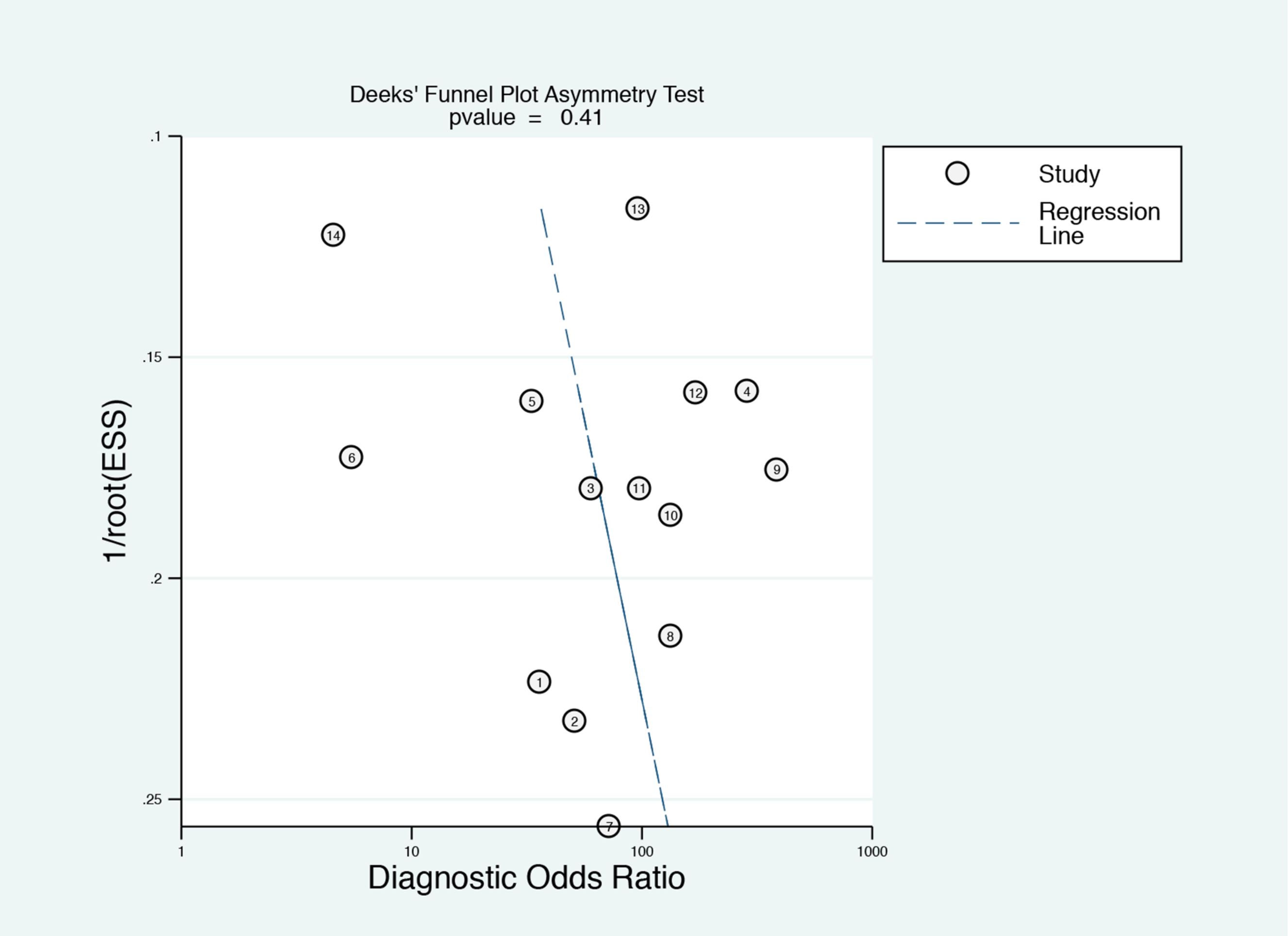

Supplementary Figure S6: Funnel graph for the assessment of potential publication bias for brain tumor subgroup.

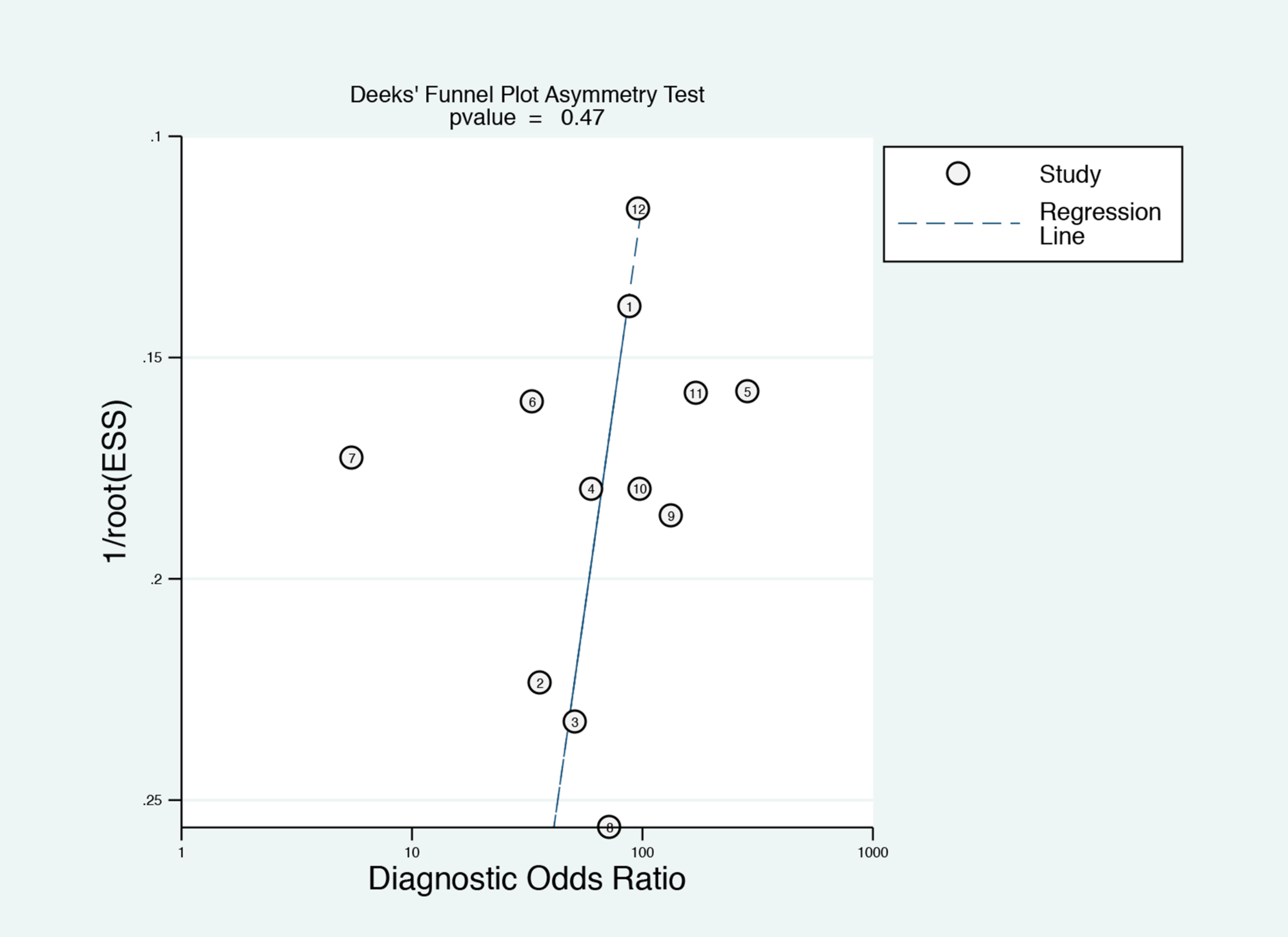

Supplementary Figure S7: Funnel graph for the assessment of potential publication bias for CSF-based miR-21 subgroup.

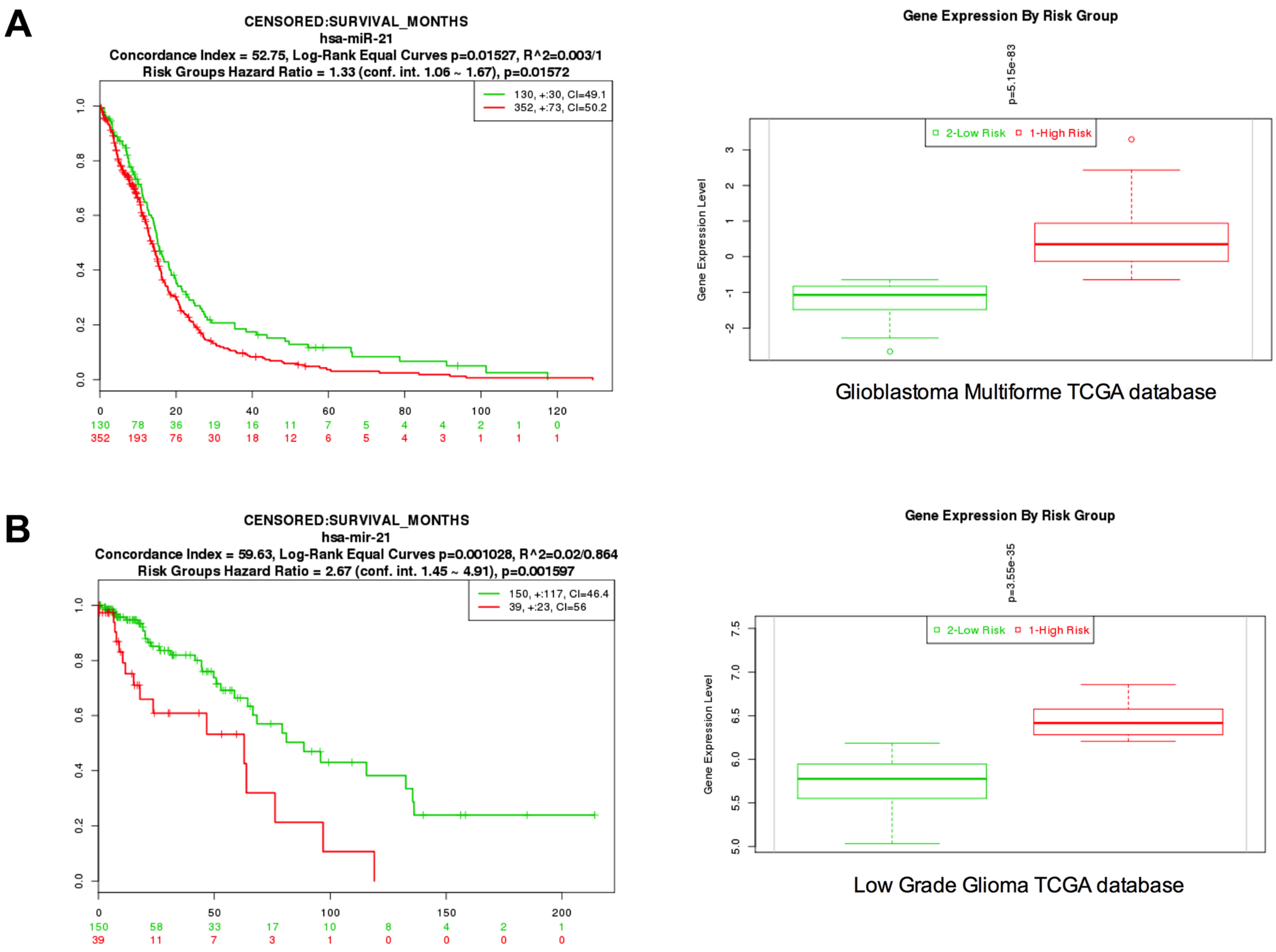

Supplementary Figure S8: Kaplan-Meier survival curves for patients with glioma and high or low miR-21 expression using TCGA databases. (**A**) Glioblastoma multiforme TCGA database; (**B**) Low grade glioma TCGA database.

Supplementary Table S1: Characteristics of diagnostic clinical trials included in the meta-analysis.
See Supplementary_Table_S1

Supplementary Table S2: Patients' clinicopathological characters in our validation cohort (*N* = 35)

Variables	Value
No. patients	35
Age, median (range), yr	52 (45–71)
Male, No. (%)	21 (60.0%)
KPS score	
≥ 80	11 (31.4)
< 80	24 (68.6)
Grade	
WHO II	9 (25.7)
WHO III	11 (31.4)
WHO IV	15 (42.9)
Recurrence, No. (%)	18 (51.4)
Death, No. (%)	27 (77.1)

KPS, Karnofsky performance status.
