# Supplementary material for: Extracellular miRNA-21 as a novel biomarker in glioma: evidence from meta-analysis, clinical validation and experimental investigations
Source: Oncotarget. 2016 May 5;7(23):33994–4010. doi: 10.18632/oncotarget.9188 (PMC5085133; doi:10.18632/oncotarget.9188)
Supplement: Supplementary file 2 [file oncotarget-07-33994-s002.docx]

Supplementary Table S1: Characteristics of diagnostic clinical trials included in the meta-analysis

| Author (year) | Ethnicity | Country | Sample size | | Tumor site | Sample source | Diagnostic tests | |
| --- | --- | --- | --- | --- | --- | --- | --- | --- |
|  |  |  | Case | Control |  |  | Sens | Spec |
| Wang (2009) | Caucasian | US | 28 | 19 | Pancreatic cancer | Plasma | 46.0 | 89.0 |
| Sadakari (2010) | Asian | Japan | 12 | 3 | Pancreatic cancer | Digestive juice | 91.7 | 100.0 |
| Tsujiura (2010) | Asian | Japan | 34 | 15 | Gastric cancer | Plasma | 60.9 | 63.3 |
| Asaga (2011) | Caucasian | US | 102 | 20 | Breast cancer | Serum | 67.3 | 75.0 |
| Baraniskin (2011) | Caucasian | Germany | 23 | 30 | Brain tumor | Cerebrospinal fluid | 95.7 | 80.0 |
| Komatsu (2011)a | Asian | Japan | 50 | 20 | Esophageal cancer 1 | Plasma | 88.0 | 70.0 |
| Komatsu (2011)b | Asian | Japan | 50 | 20 | Esophageal cancer 2 | Plasma | 48.2 | 84.7 |
| Li (2011) | Asian | China | 20 | 10 | Lung cancer | Blood | 78.8 | 100.0 |
| Ryu (2011) | Asian | Korea | 24 | 16 | Pancreatic cancer | Digestive juice | 76.0 | 80.0 |
| Shen (2011) | Caucasian | US | 58 | 29 | Lung cancer | Plasma | 79.3 | 65.5 |
| Wei (2011) | Asian | China | 77 | 36 | Lung cancer | Plasma | 61.0 | 83.3 |
| Xu (2011) | Asian | China | 101 | 89 | HCC | Serum | 84.0 | 73.5 |
| Zheng (2011) | Asian | China | 53 | 20 | Gastric cancer | Plasma | 83.8 | 80.5 |
| Liu (2011)a | Asian | China | 45 | 45 | Pancreatic cancer 3 | Plasma | 66.7 | 71.1 |
| Liu (2011)b | Asian | China | 45 | 30 | Pancreatic cancer 4 | Plasma | 77.8 | 66.7 |
| Kanaan (2012) | Caucasian | US | 50 | 50 | Colorectal cancer | Plasma | 90.0 | 90.0 |
| Le (2012) | Asian | China | 82 | 50 | Lung cancer | Serum | 46.3 | 92.0 |
| Li (2012) | Asian | China | 70 | 70 | Gastric cancer | Plasma | 74.3 | 75.7 |
| Liu (2012) | Asian | China | 57 | 59 | HCC | Serum | 89.5 | 71.2 |
| Teplyuk (2012)a | Caucasian | Germany | 19 | 15 | Glioblastoma | Cerebrospinal fluid | 57.9 | 80.0 |
| Teplyuk (2012)b | Caucasian | Germany | 16 | 15 | Metastatic brain tumor from breast cancer | Cerebrospinal fluid | 93.8 | 80.0 |
| Teplyuk (2012)c | Caucasian | Germany | 26 | 15 | Metastatic leptomeningeal tumor from breast cancer | Cerebrospinal fluid | 100.0 | 86.7 |
| Teplyuk (2012)d | Caucasian | Germany | 28 | 15 | Metastatic brain tumor from lung cancer | Cerebrospinal fluid | 89.3 | 80.0 |
| Teplyuk (2012)e | Caucasian | Germany | 4 | 15 | Metastatic leptomeningeal tumor from lung cancer | Cerebrospinal fluid | 75.0 | 100.0 |
| Tomimaru (2012)a | Asian | Japan | 126 | 30 | HCC 3 | Plasma | 61.1 | 83.3 |
| Tomimaru (2012)b | Asian | Japan | 126 | 50 | HCC 4 | Plasma | 87.3 | 92.0 |
| Wang (2012)a | Asian | China | 50 | 39 | Breast cancer | Serum | 80.0 | 87.7 |
| Wang (2012)b | Asian | China | 32 | 39 | Colorectal cancer | Serum | 87.5 | 74.4 |
| Wang (2012)c | Asian | China | 31 | 39 | Lung cancer | Serum | 87.1 | 74.4 |
| Wang (2012)d | Asian | China | 30 | 39 | Gastric cancer | Serum | 56.7 | 94.9 |
| Wang (2012)e | Asian | China | 31 | 39 | Esophageal cancer | Serum | 71.0 | 69.2 |
| Wang (2012)f | Asian | China | 10 | 10 | Brain tumor | Plasma | 90.0 | 100.0 |
| Wang (2012)g | Asian | China | 30 | 10 | Glioma | Plasma | 100.0 | 90.0 |
| Abd-El-Fattah (2013) | African | Egypt | 65 | 37 | Lung cancer | Serum | 85.7 | 86.5 |
| Akers (2013)a | Caucasian | US | 13 | 14 | Brain tumor 5 | Cerebrospinal fluid | 85.0 | 100.0 |
| Akers (2013)b | Caucasian | US | 15 | 16 | Brain tumor 6 | Cerebrospinal fluid | 87.0 | 93.0 |
| Cui (2013) | Asian | China | 42 | 99 | Gastric cancer | Digestive juice | 85.7 | 97.8 |
| Egidi (2013) | Caucasian | Italy | 38 | 40 | Prostate cancer | Serum | 52.6 | 72.5 |
| Gao (2013) | Asian | China | 89 | 55 | Breast cancer | Serum | 87.6 | 87.3 |
| Kishimoto (2013)a | Asian | Japan | 94 | 23 | Biliary tract cancer 3 | Plasma | 72.3 | 91.3 |
| Kishimoto (2013)b | Asian | Japan | 94 | 50 | Biliary tract cancer 4 | Plasma | 85.1 | 100.0 |
| Kumar (2013) | Asian | India | 14 | 8 | Breast cancer | Plasma | 92.9 | 100.0 |
| Liu (2013)a | Asian | China | 200 | 80 | Colorectal cancer | Serum | 65.0 | 85.0 |
| Liu (2013)b | Asian | China | 217 | 73 | Head&neck cancer | Plasma | 76.0 | 69.9 |
| Luo (2013) | Caucasian | Germany | 80 | 144 | Colorectal cancer | Plasma | 51.7 | 80.7 |
| Mar-Aguilar (2013) | Latinos | Mexico | 61 | 10 | Breast cancer | Serum | 94.4 | 80.0 |
| Mozzoni (2013) | Caucasian | Italy | 54 | 46 | Lung cancer | Plasma | 70.0 | 62.5 |
| Ouyang (2013) | Asian | China | 80 | 80 | Osteosarcoma | Plasma | 76.5 | 97.0 |
| Que (2013) | Asian | China | 22 | 27 | Pancreatic cancer | Serum | 95.5 | 81.5 |
| Si (2013) | Asian | China | 52 | 20 | Breast cancer | Serum | 79.0 | 100.0 |
| Tang (2013)a | Asian | China | 62 | 60 | Lung cancer | Plasma | 48.4 | 78.3 |
| Tang (2013)b | Asian | China | 34 | 32 | Lung cancer | Plasma | 52.9 | 71.9 |
| Toiyama (2013)a | Asian | Japan | 186 | 53 | Colorectal cancer | Serum | 82.8 | 90.6 |
| Toiyama (2013)b | Asian | Japan | 43 | 53 | Advanced colorectal adenoma | Serum | 76.7 | 81.1 |
| Xie (2013) | Asian | China | 39 | 19 | Esophageal cancer | Digestive juice | 89.7 | 47.4 |
| Ng(2013) | Asian | China | 170 | 100 | Breast cancer | Plasma | 75.3 | 78.0 |
| Ma (2013) | Caucasian & African | US | 36 | 38 | Lung cancer | Plasma | 75.0 | 76.3 |
| Abue (2014)a | Asian | Japan | 32 | 30 | Pancreatic cancer 4 | Plasma | 75.0 | 73.0 |
| Abue (2014)b | Asian | Japan | 32 | 42 | Pancreatic cancer 3 | Plasma | 80.0 | 63.0 |
| Du (2014) | Asian | China | 49 | 49 | Colorectal cancer | Plasma | 76.2 | 93.2 |
| Jones (2014) | Caucasian | Australia | 42 | 20 | Hodgkin lymphoma | Plasma | 95.0 | 86.0 |
| Kotb (2014) | African | Egypt | 10 | 10 | Prostate cancer | Serum | 90.0 | 90.0 |
| Shen (2014) | Caucasian | US | 64 | 73 | Lung cancer | Digestive juice | 53.1 | 55.9 |
| Wang (2014) | Asian | China | 52 | 49 | Head&neck cancer | Serum | 69.2 | 81.6 |
| Zhang (2014) | Asian | China | 41 | 30 | Colorectal cancer | Plasma | 51.2 | 79.0 |
| Yuan (2014) | Asian | China | 20 | 20 | Glioma | Cerebrospinal fluid | 90.0 | 95.0 |
| Mao (2014) | Asian | China | 56 | 122 | CNS lymphoma | Serum | 85.7 | 90.2 |
| Liu (2014) | Asian | China | 65 | 65 | Retinoblastoma | Plasma | 46.2 | 65.3 |
| Ye (2014)a | Asian | China | 100 | 50 | Esophageal cancer | Plasma | 97.0 | 56.0 |
| Ye (2014)b | Asian | China | 100 | 50 | Esophageal cancer | Saliva | 89.0 | 64.0 |
| Zanutto (2014) | Caucasian | Italy | 29 | 29 | Colorectal cancer | Plasma | 58.6 | 58.6 |
| Ogata-Kawata (2014) | Asian | Japan | 88 | 11 | Colorectal cancer | Serum | 61.4 | 90.9 |
| Basati (2014) | Asian | Iran | 40 | 40 | Colorectal cancer | Serum | 77.5 | 77.5 |
| Wu (2015)a | Asian | China | 50 | 50 | Gastric cancer | Serum | 88.0 | 80.0 |
| Wu (2015)b | Asian | China | 50 | 50 | Gastric cancer | PBMC | 82.0 | 74.0 |
| Erbes (2015) | Caucasian | Germany | 24 | 24 | Breast cancer | Urine | 66.7 | 91.7 |
| Humeau (2015) | Caucasian | France | 7 | 4 | Pancreatic cancer | Saliva | 71.4 | 100.0 |
| Shi (2015)a | Asian | China | 70 | 25 | Glioma | Cerebrospinal fluid | 80.0 | 96.0 |
| Shi (2015)b | Asian | China | 50 | 25 | Glioma | Serum | 64.0 | 72.0 |

1. Normalize by miR375; 2. Normalize by RNU6B; 3. Patients with non-tumor benign disease as control; 4. Healthy volunteer as control; 5. Training study; 6. Validation study.
